# Biodegradation of weathered polystyrene films in seawater microcosms

**DOI:** 10.1038/s41598-017-18366-y

**Published:** 2017-12-21

**Authors:** Evdokia Syranidou, Katerina Karkanorachaki, Filippo Amorotti, Martina Franchini, Eftychia Repouskou, Maria Kaliva, Maria Vamvakaki, Boris Kolvenbach, Fabio Fava, Philippe F.-X. Corvini, Nicolas Kalogerakis

**Affiliations:** 1 School of Environmental Engineering, Technical University of Crete, Chania, Greece; 2 School of Mineral Resources Engineering, Technical University of Crete, Chania, Greece; 3 Department of Materials Science & Technology, University of Crete, Heraklion, Greece; 4 Institute for Ecopreneurship, School of Life Sciences, FHNW, Switzerland; 5 Department of Civil, Chemical, Environmental and Materials Engineering (DICAM), University of Bologna, Bologna, Italy

## Abstract

A microcosm experiment was conducted at two phases in order to investigate the ability of indigenous consortia alone or bioaugmented to degrade weathered polystyrene (PS) films under simulated marine conditions. Viable populations were developed on PS surfaces in a time dependent way towards convergent biofilm communities, enriched with hydrocarbon and xenobiotics degradation genes. Members of Alphaproteobacteria and Gammaproteobacteria were highly enriched in the acclimated plastic associated assemblages while the abundance of plastic associated genera was significantly increased in the acclimated indigenous communities. Both tailored consortia efficiently reduced the weight of PS films. Concerning the molecular weight distribution, a decrease in the number-average molecular weight of films subjected to microbial treatment was observed. Moreover, alteration in the intensity of functional groups was noticed with Fourier transform infrared spectrophotometry (FTIR) along with signs of bio-erosion on the PS surface. The results suggest that acclimated marine populations are capable of degrading weathered PS pieces.

## Introduction

Plastic debris have been characterized as a major pollutant in the marine environment^1,2^ since their input volumes have been estimated in the order of million tons per year^1^. The buoyant fraction is widely distributed in oceans and seas worldwide but the vast majority is concentrated in the subtropical gyres^3,4^. Next to gyres, the Mediterranean Sea represents a significant accumulation zone of floating plastic litter with an average density of 1 plastic item per 4 m^2^ ^5^. Ruiz-Orejón *et al*.^6^ estimated that 1455 tons of debris are floating in Mediterranean and pieces with 1 mm^2^ surface area are the most abundant. Moreover, recent studies revealed that elevated concentrations of plastic load were detected in surface waters close to coastline^7,8^.

Polystyrene (PS) is among the most commonly used plastics; it is frequently found in the environment as a material from diverse uses such as packaging foams and disposable cups^9^. Its demand only, reached the 7% of total polymer demand in Europe in 2014, which can be translated into approximately 4 million tons^10^. Since it is mainly used for manufacturing of single-use products, a large portion of post-consumer production ends up into landfills or into oceans^2,10^ and remains there for several hundred years due to their persistence to degradation.

In the marine environment, immersed or floating plastics are rapidly colonized by pioneer microbial species^11,12^. These efficient colonizers develop diverse and distinct communities from the surrounding planktonic populations^13^ and are equipped with genes related to biofilm lifestyle^14^. The composition of the adhered assemblages depends on the season, location and the type of plastic^15^. With respect to the effect of the polymer, it is still not clear whether the type of plastic can strongly drive the divergence of microbial communities. For example, differences were detected between two biofilm diatom communities colonizing polyethylene and biodegradable polymer after 33 days of exposure^16^. Whereas, known biofilm species attach the surface of polyethylene terephthalate (PET) bottles; no significant differences were found between PET associated communities and either glass or particle associated communities^17^. Hence, interactions between polymer and the adhered community are yet to be elucidated^18^.

This is very crucial in microbe-mediated degradation of plastics since biofilm formation is considered as the first step of the process^19^. Biodeterioration initiates when the microbes attach the polymer surface using physical, chemical or enzymatic ways^20^. Changes on the surface topography such as the presence of cracks and fissures have been attributed to associated biota^21,22^. Next, bio-fragmentation in terms of producing smaller molecular weight products and assimilation follows^20^. Most of the studies present indirect signs of polymer biodegradation^23,24^ and only very few have demonstrated the consumption of the polymer through the detection of monomers in the medium or through monitoring the fate of the labeled polymer^25,26^.

Taking into consideration that the vast majority of plastic colonizers is still unexplored, there is a high chance to unravel species or consortia with suitable catalytic characteristics. With the aim to explore the ability of indigenous marine consortia to degrade naturally weathered PS films, a two-phase microcosm experiment was conducted. During the first phase, indigenous marine population alone or bioaugmented with strains able to grow with weathered PS as the sole carbon source were incubated with naturally weathered PS pieces in order to investigate for plastic colonizing microbes and their potential impact on PS degradation. In the second phase, the acclimated biofilm communities were again incubated with weathered PS pieces to test whether there is any enhancement in PS degradation. At the same time, significant changes on the response of biofilm community was monitored.

## Results

In this study, a two-phase microcosm experiment was performed in order to investigate whether two different marine consortia could degrade naturally weathered PS pieces. The indigenous marine community itself (treatment “INDG”) and bioaugmented with PS competent strains (treatment “BIOG”) were incubated with PS films under oligotrophic conditions, where polystyrene was the only available carbon source and enriched seawater was used as the aqueous medium. At the end of six months (phase I), the developed biofilm was harvested and re-incubated with weathered PS films under same environmental conditions for another six months (phase II).

### Planktonic and biofilm populations

In the beginning of each phase, PS pieces were sterilized to ensure that only members of the studied communities would grow. During phase I, biofilm formation was detected on all PS sample pieces (from both INDG and BIOG treatments) by naked eye after 4 months of incubation, despite the unavailability of an easily accessible carbon source. At end of phase I, a fraction of the planktonic and the attached assemblages was cultured in rich medium to verify the microbial survival. With respect to BIOG treatment, 10^2^ CFU ml^−1^ and approximately 10^3^ CFU cm^−2^ were enumerated in samples from aqueous medium and biofilm matrix respectively. Considering the initial concentration of inoculated population, a decrease in cell abundances was noticed after 6 months incubation. Higher free cell densities were observed in the INDG treatment at the end of phase I (free cells: 10^4^ CFU ml^−1^ and biofilm: 10^3^ CFU cm^−2^).

**Figure 1:**
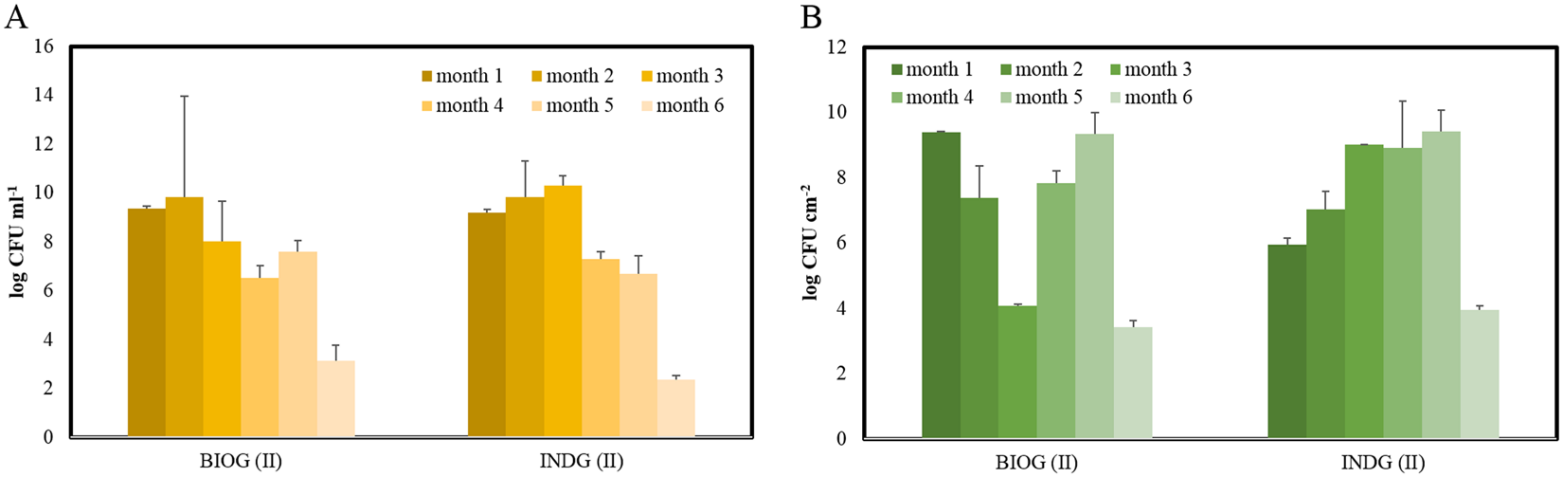
Abundances of (**A**) free cells in the seawater medium and (**B**) abundances of the biofilm cells on the PS pieces during phase II. Bars indicate standard deviation.

At phase II, monthly samplings were conducted in order to estimate the growth trend of the planktonic population and the microbial biofilm established on the weathered polymer’s surface. As seen in Fig. 1A, both planktonic populations exhibited the same pattern. They reached a maximum cell density at the end of month 2 (BIOG: 10^9^ CFU ml^−1^, INDG: 10^11^ CFU ml^−1^) and then cell densities progressively decreased until month 6. Two-way ANOVA revealed no significant effect of month (F: 0.6, p > 0.05) or treatment (F:1.5, p > 0.05) as well as no interaction effect (F: 0.5, p > 0.05) was verified.

Interestingly, the two acclimated consortia also displayed a similar pattern of polymer colonization (Fig. 1B). Microbes efficiently adhered to the weathered PS films and developed a population from the first month, since 10^6^ CFU cm^−2^ were measured. They continued to grow and the highest abundances were observed at the end of month 4 (BIOG: 10^10^ CFU cm^−2^, INDG: 10^10^ CFU cm^−2^). At this time interval, the biofilm concentrations were significantly different with all the previous concentrations measured. Afterwards, a decrease was noticed and cell densities from both treatments were reduced to approximately 10^4^ CFU cm^−2^ at the end of month 6. Again, no significant effects were detected due to “month” (F: 1.5, p > 0.05), or “treatment” (F: 0.08, p > 0.05) or interaction effect (F: 0.05, p > 0.05).

### Weight loss

**Figure 2:**
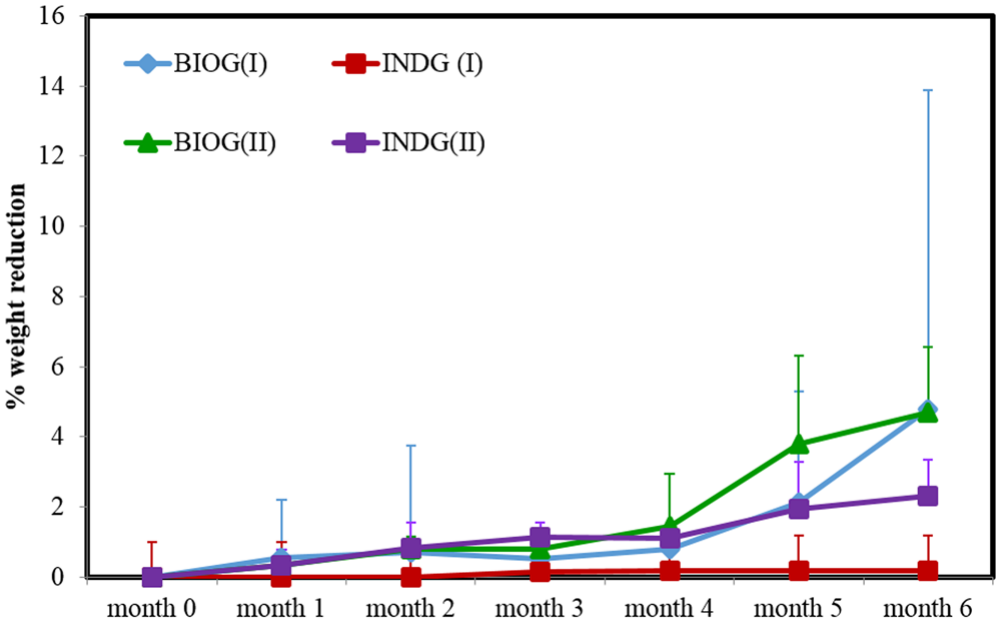
Percentage of weight reduction by the different marine consortia (I: phase I, II: phase II). Bars indicate standard deviation.

The weight reduction due to biodegradation is shown in Fig. 2. The BIOG consortia efficiently reduced by 0.5% the mass of PS pieces after one month of incubation during phase I. During the following months, the weight decline continued and it reached 4.7% after 6 months incubation. When the acclimated consortia were employed in phase II, no higher level of biodegradation was observed since a 4.7% reduction was also measured at the end of phase II. When the indigenous community was used (treatment INDG), almost no weight reduction was detected at the end of phase I, as only a 0.19% weight loss was measured at month 6. The re-inoculation of biofilm population to seawater containing weathered PS as the sole carbon source in phase II enhanced the weight reduction. The percentage of weight loss reached 0.3% after 1 month of incubation and it kept increasing throughout the experimental period. The highest weight loss (2.3%) was recorded at month 6. Two-way ANOVA demonstrated significant effect due to “treatment” (F: 3.8, p = 0.05) but no significant effect due to “month” (F: 0.07, p > 0.05) or interaction effect (F: 0.01, p > 0.05).

### SEM analysis

**Figure 3:**
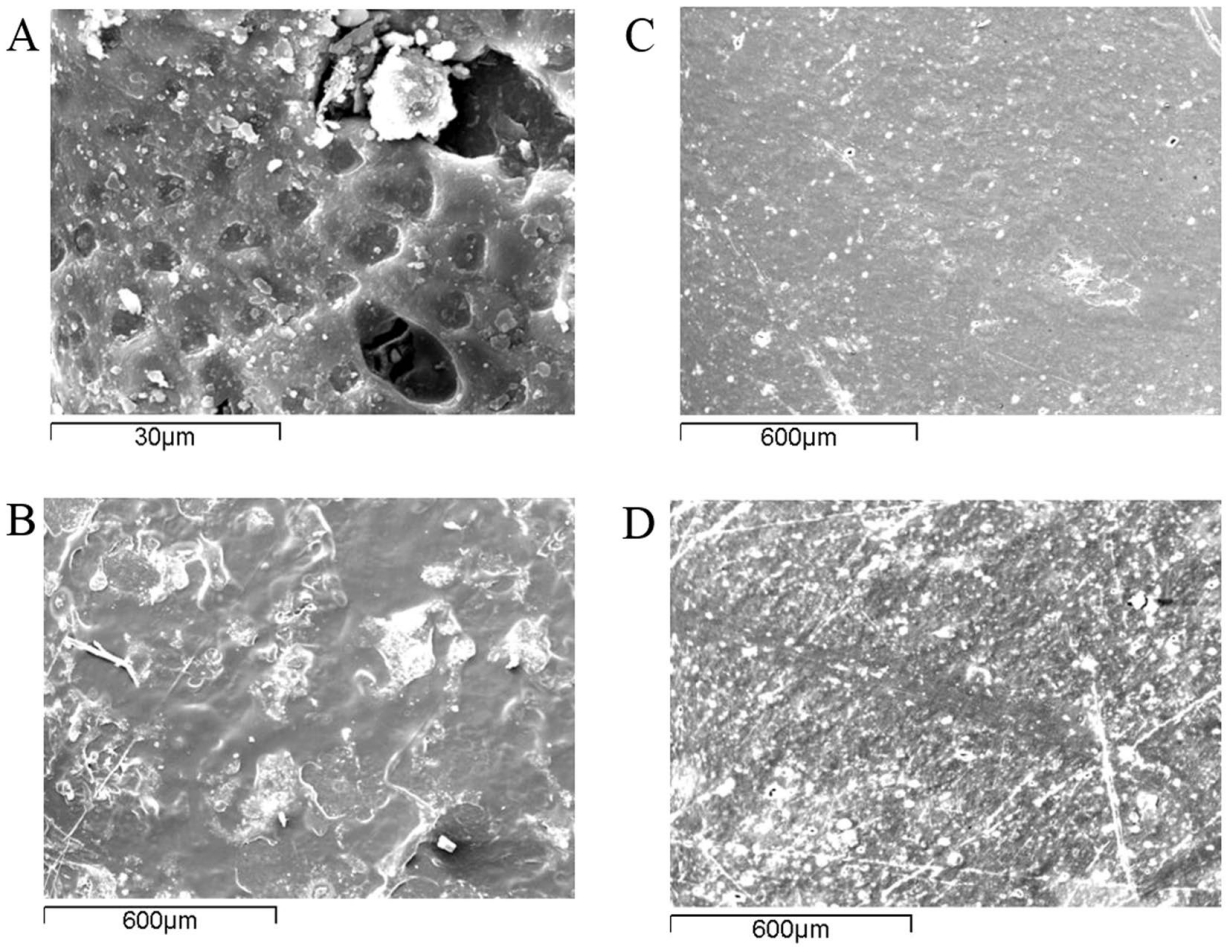
SEM images of biofilm formed by the marine microbes on polystyrene after 1 month incubation (**A**) and after 2 months incubation (**B**). SEM images showing the surface modifications of the weathered pieces before (**C**) and after (**D**) the microbial treatment at the end of the experiment and after removal of the biofilm.

Biofilm succession and changes on the surface topography of the PS films were visualized with scanning microscopy. As seen in Fig. 3A, a dense biofilm matrix was formed on the pieces after only one month of incubation. The PS surfaces were fully covered and the biofilm was thick and compact after two months of incubation (Fig. 3B).

The comparison of untreated and microbially treated PS films revealed differences on the polymers’ surface. The naturally weathered samples had smoother surfaces without any significant cracks or holes despite the fact that they were previously exposed to physical/mechanical treatment (i.e. waves, temperature and UV irradiation) (Fig. 3C). When incubated with the marine consortia, many fissures were detected throughout the surface and the roughness increased as shown in Fig. 3D once the biofilm was removed. In particular, these PS films bear cracks and a rough surface.

### GPC analysis

The residual polymer was further analyzed with gel-permeation chromatography (GPC) in order to detect any potential oligomers and any shift of the molecular weight distribution. As seen in Table 1, the weight-average molecular weight (M_w_) of the PS pieces remains constant after being exposed to microbial consortia although a decrease in the number-average molecular weight (M_n_) can be observed after month 2 for both treatments. At the end of phase II, the M_n_ of the weathered PS films decreased by 32% and by 30.5% in BIOG and INDG treatment, respectively. The polydispersivity (M_w_/M_n_) of the weathered pieces exhibited lower values than the microbially treated pieces. When the GPC profiles of PS pieces collected at different time intervals was compared, no significant differences were observed in the main peak, however a tail in the longer retention times was observed suggesting the formation of lower molecular weight species which also led to the broadening of the molecular weight distribution discussed above (Fig. S1).

**Table 1:** Molecular weights and molecular weight distribution of the naturally weathered and microbially treated PS pieces.

Month	Treatment	*M*_n_	*M*_w_	*M*_w_/*M*_n_
0	Weathered PS	115,800 ± 13900	165,600 ± 7700	1.43
1	BIOG	115,600 ± 6400	164,500 ± 5100	1.42
1	INDG	103,800 ± 5300	154,300 ± 2000	1.49
2	BIOG	80,400 ± 11000	161,900 ± 81000	2.02
2	INDG	83,500 ± 6600	163,600 ± 200	1.97
4	BIOG	83,200 ± 12006	163,200 ± 12500	1.97
4	INDG	86,000 ± 10400	173,000 ± 13400	2.02
6	BIOG	78,500 ± 5100	157,900 ± 1900	2.01
6	INDG	80,400 ± 4900	168,400 ± 600	2.10

### Chemical changes in PS surface

**Figure 4:**
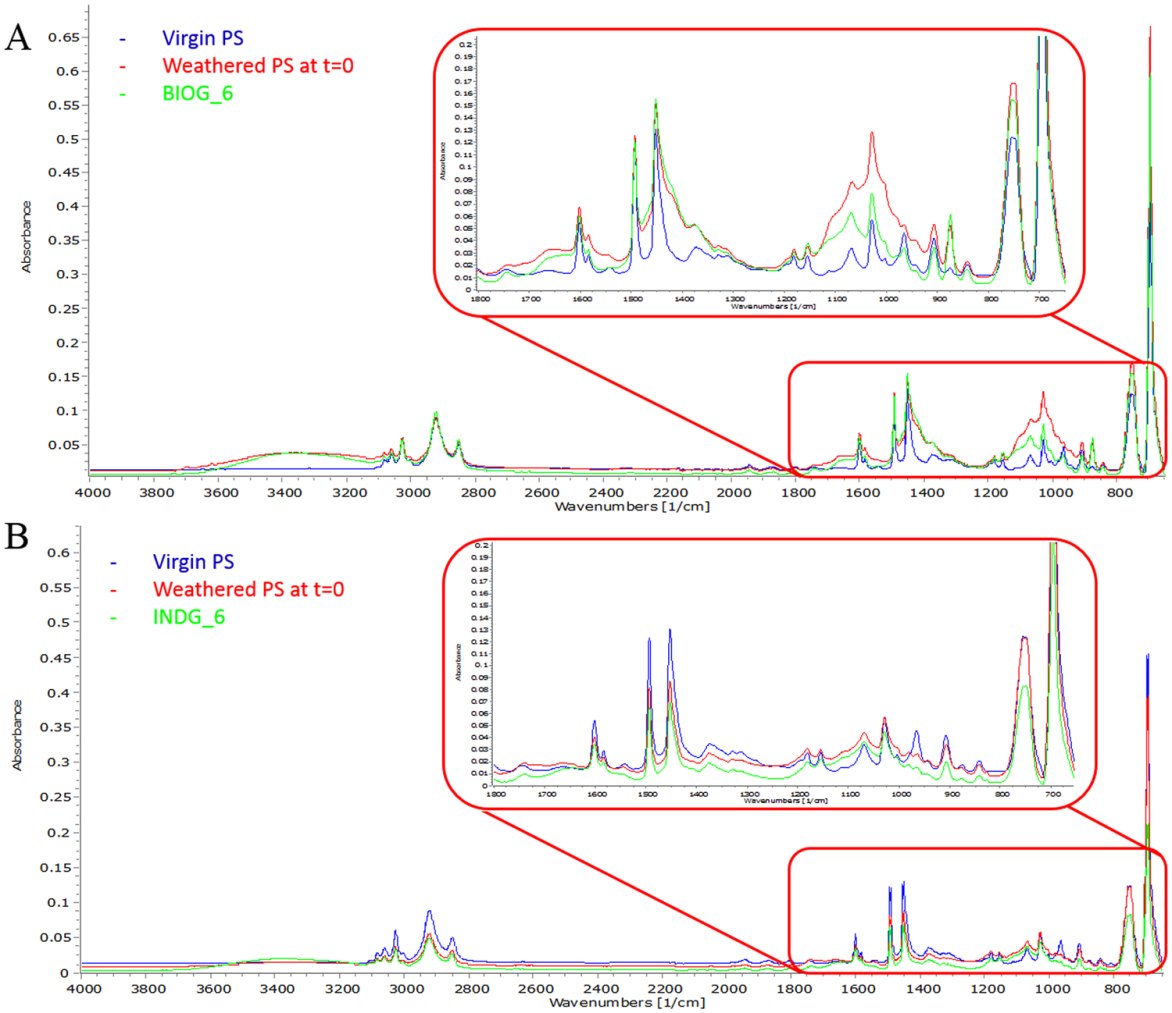
FTIR spectra of virgin, naturally weathered (at t = 0) and microbially treated PS films by the BIOG consortium at the end of 6 months (**A**) and microbially treated PS films by the INDG consortium at the end of 6 months.

The FTIR spectra of virgin, naturally weathered (prior to microbial treatment) and microbially treated PS films are depicted in Fig. 4Α and Β. The PS bands shared in all samples are at 3025, 2921, 2850, 1600, 1492, 1451, 1068, 1028, 965, 907, 755 and 695 cm^−1^ wavenumber. In weathered and microbially treated films, the intensity of bands altered depending on the type of degradation and the consortium they are exposed to. For example, the intensity of the band at 1600 cm^−1^ which corresponds to conjugated carbon double bonds, increased in the BIOG treated PS films and decreased in the INDG treated PS films in comparison to the virgin PS films. The peaks at 1492 and 1451 cm^−1^ correspond to the deformational vibrations of both –CH_2_ and (B_1_) of the benzene ring of the styrene molecule. A decrease in the intensity of double bonds (907–965 cm^−1^ region) was observed in microbially treated samples in comparison to the virgin ones. Interestingly, new bands with a low intensity appeared in the microbially treated films.

### PS associated community composition

**Figure 5:**
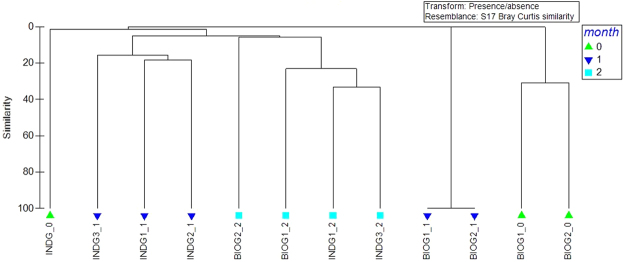
Cluster analysis based on Bray–Curtis similarities from ARISA fingerprints of marine biofilm communities on the polystyrene pieces during phase II.

The biofilm community composition during the early stages of colonization was investigated during phase II (Fig. 5A). It seems that the microbial phylotypes were clustered by successional stages (ANOSIM R: 0.404, p < 0,01). Samples that belonged to the same sampling time and treatment were grouped together. At the end of month 2, all the PS associated communities were clustered together regardless of the treatment. The factor “type of inoculated consortium” did not separate the samples.

**Figure 6:**
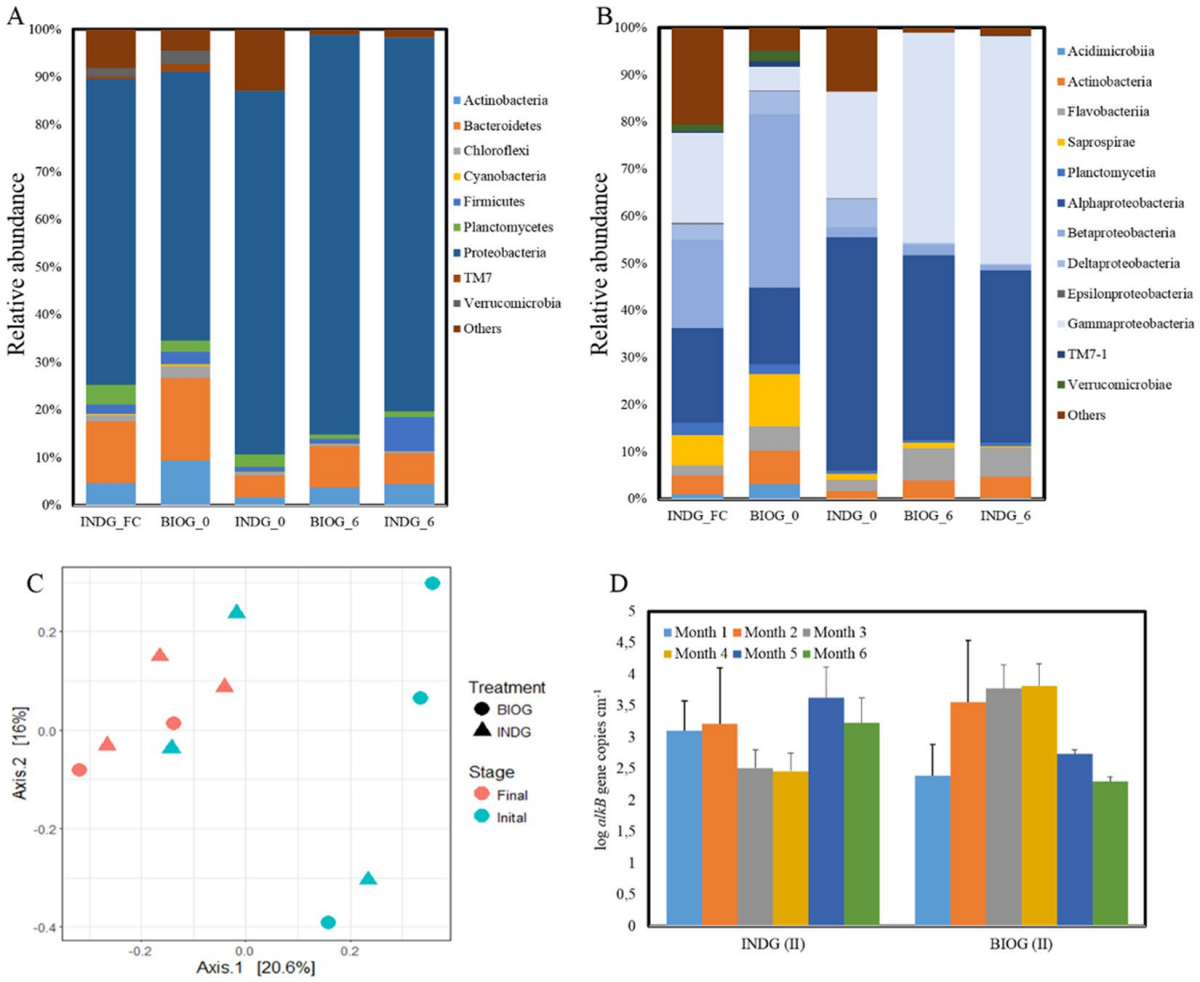
Community composition of major bacterial phyla (**A**) and classes (**B**) of the pelagic (INDG_FC) and biofilm communities in the beginning (BIOG_0 and INDG_0) and at the end (BIOG_6 and INDG_6) of phase II, (**C**) PCoA plot and (**D**) the abundances of *alkB* gene in biofilm communities during phase II. Bars indicate standard deviation.

The biofilm communities at the end of both phases I and II were compared employing next generation techniques (16S rRNA gene sequencing using the MiSeq platform). It appears that the associated communities contained several hundreds of operational taxonomic units (OTUs). In total, 48 phyla were identified, containing more than 500 bacterial genera. In all samples, Proteobacteria was the dominant phylum, though its relative abundance increased in biofilm communities established during phase II (Fig. 6A). It accounted for more than 50% in the planktonic and initial biofilm populations, while 80% of the sequences on the average were affiliated with Proteobacteria in the assemblages at the end of month 6. Within this phylum, a decrease in the abundances of Betaproteobacteria and Deltaproteobacteria was noticed between the phase I BIOG consortium (more than 30% and 4% in average respectively) and biofilm at the end of phase II (less than 1% in average) (Fig. 6B). Whereas the abundances of Alphaproteobacteria and Gammaproteobacteria increased in these samples.

Bacteroidetes (16%) was the second most abundant population in initial samples, followed by Actinobacteria (9%) and Verrucomicrobia (3%), while these phyla were less represented in the phase II biofilm communities. In accordance, the order Actinobacteria, Saprospirae and Verrucomicrobiae exhibited a decrease in these assemblages while the order Flavobacteriia increased. The abundances of inoculated genera *Shewanella*, *Rhodococcus* and *Pseudomonas* were at similar levels in all samples.

A PCoA plot was generated using the matrix of unweighted Unifrac distances (Fig. 6C). It revealed that the initial communities were clustered and were distinct from the final biofilm communities (ANOSIM R: 0.32, p = 0.073), when time was used as a factor. Whereas, the treatment as a factor could not separate the adhered assemblages, demonstrating that the indigenous species dominated the bioaugmented biofilm communities.

The abundances of *alkB* gene within the acclimated biofilm communities were monitored during phase II (Fig. 6D). It seems that the concentration of this gene displayed variations depending on the time of sampling, although these variations were not statistically significant according to two-way ANOVA (F: 0.29, p > 0.05). The highest number of *alkB* gene copies was observed at the end of month 5 in ING communities in line with the population density. Concerning BIOG assemblages, the *alkB* abundance increased with respect to time until the end of month 4 and then progressively decreased.

**Figure 7:**
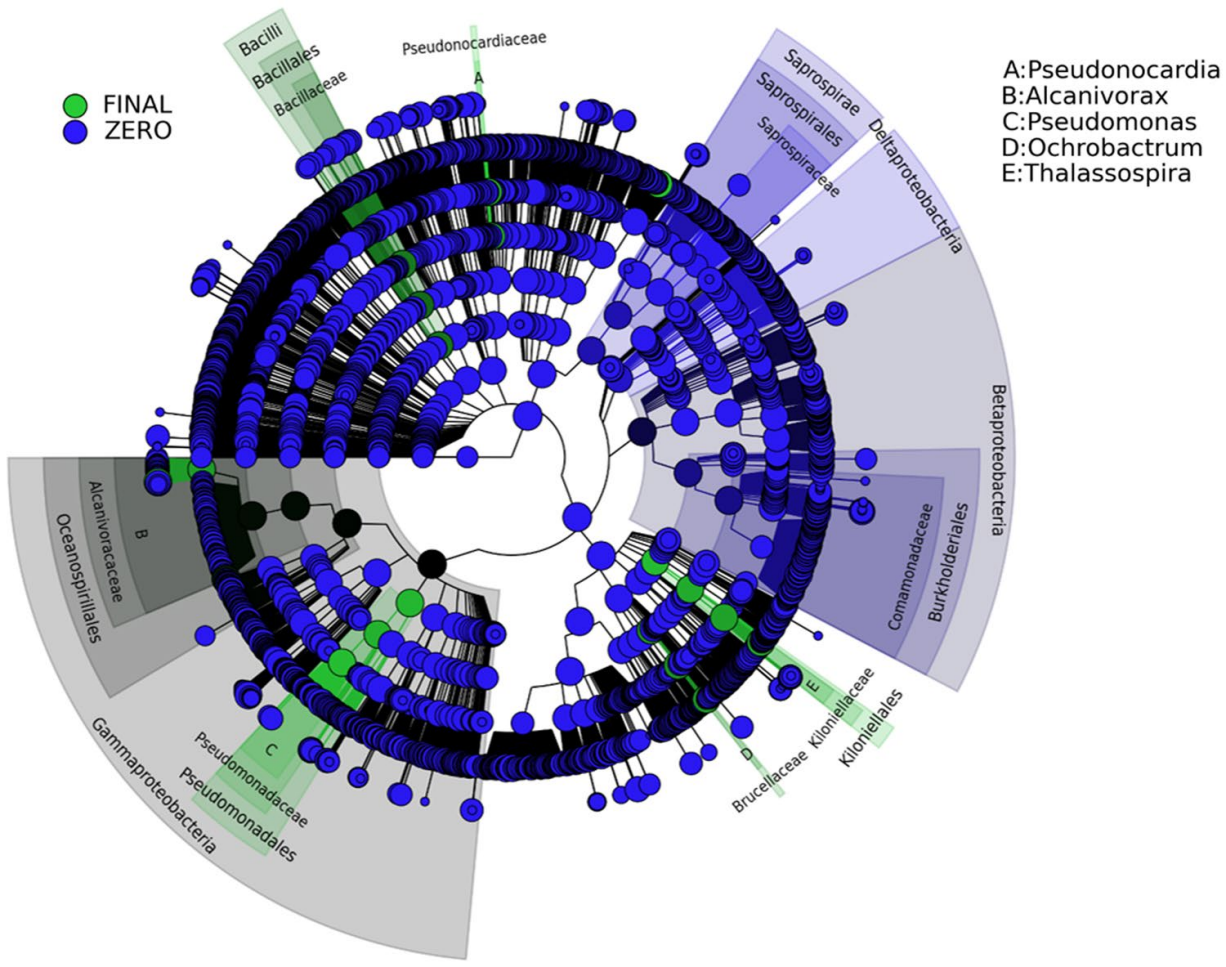
Biofilm biomarkers of the initial INDG consortium and the final developed communities. LEfSe was used to validate the statistical significance and the effect size of the differential abundances of taxa (Kruskal-Wallis and Wilcoxon rank-sum p < 0.05 and LDA score >4). In the cladogram, the class, order and family are represented and the genus is represented using letters.

**Figure 8:**
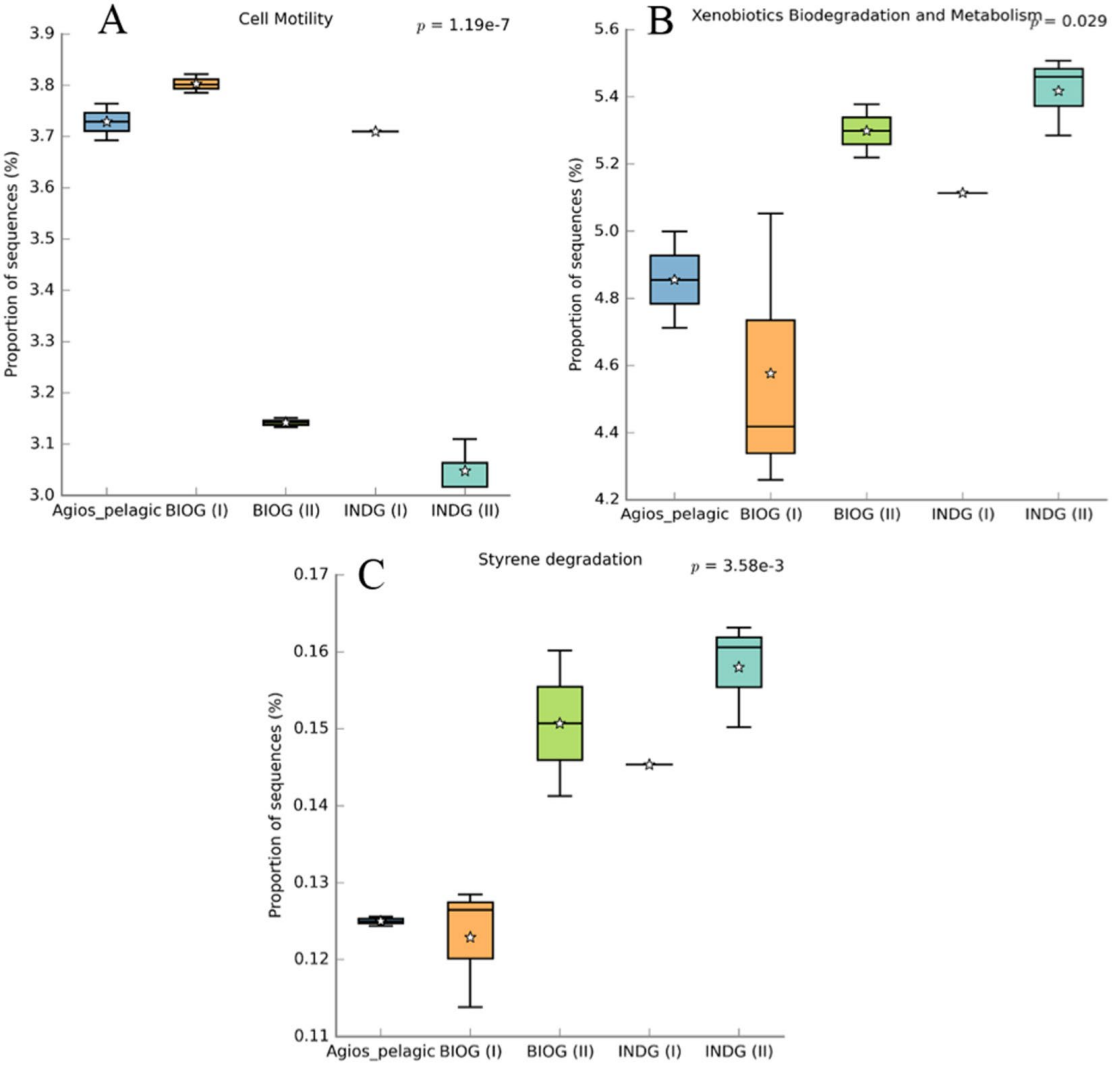
Predicted abundances of enzyme-encoding genes involved in (**A**) cell motility, (**B**) xenobiotic degradation and metabolism and (**C**) styrene degradation. Bars indicate standard deviation.

The Linear Discriminant Analysis Effect Size (LEfSe) was used to identify the species-biofilm biomarkers for initial consortium (end of phase I) versus final acclimated communities (phase II) (Fig. 7). It revealed that 32 OTUs were found to be discriminant within the INDG community (LEfSe p < 0.05, log10 LDA-score >4). Members of Betaproteobacteria, Deltaproteobacteria and Saprospirae were highly represented in the INDG adhered assemblages at the end of phase I. In the acclimated INDG populations, OTUs belonged to the families *Kiloniellaceae*, *Alcanivoracaceae*, *Brucellaceae*, *Flavobacteriaceae*, *Pseudomonadaceae*, *Bacillaceae* and *Pseudonocardiaceae* were significantly enriched. Despite the difference between the non-acclimated and acclimated BIOG communities, no distinctive members were identified. The analysis of the predicted functional diversity of the biofilm communities revealed that a significant fraction of the adhered communities developed at the end of phase II preferred the sessile way of life (Fig. 8A). Moreover, the abundances of genes associated with metabolism and xenobiotics and styrene degradation increased significantly in these adhered populations (Fig. 8B and C).

## Discussion

Surfaces deployed in oceans may serve as an attractive habitat; they adsorb dissolved organic molecules and provide a substrate to various microorganisms^27^. Similarly, once plastics are transferred to oceans they may undergo biofouling. Many factors such as the available surface area, the size and the surface to volume ratio of a polymer strongly influence its susceptibility to biofouling^28^ together with the type of polymer and the prevailing environmental conditions^29^. A high diversity of marine prokaryotic and eukaryotic organisms such as diatoms, bacteria and bryozoans could inhabit plastic debris and may contribute to polymer degradation^16,21^. In general, plastics are considered resistant to microbial degradation due to their internal properties such as the high C/N ration, the macromolecular structure and the type of functional goups^30,31^.

PS is considered highly resistant to enzymatic activity and it contains phenyl side groups in a disorder manner, hence PS biodegradation is a particularly slow process^32^. When PS flakes were incubated with the strain *Rhodococcus ruber* C208 in a synthetic medium, 0.5% and 0.8% weight reduction was noticed after 4 and 8 weeks respectively^33^. The addition of pro-oxidants and subjection to weathering may be implemented as strategies to enhance PS biodegradation^32^. Under aerobic conditions, protons are abstracted from polystyrene chain and oxygen is added due to exposure to UV radiation, leading to *β*-scission^34^. In this context, naturally weathered PS pieces were used in this experiment in order to investigate the potential of marine consortia to efficiently reduce the weight of PS films. At the end of phase II, the BIOG community decreased by 4.7% the PS mass whereas the INDG community decreased the PS films by 2.3%.

Gravimetric measurements provide the first insights about the efficiency of the exploited microorganisms to degrade plastics, however, further tests are needed to verify polymer degradation^30^. Gel permeation chromatography is commonly implemented to determine potential decrease in the molecular mass^35,36^. In this experiment, the comparison of GPC profiles of weathered PS films against the microbially treated films revealed no significant differences in the main polymer peak. This pattern is expected when biodegradation occurs only on the surface of the polymer^25^ since most of the low molecular weight fragments are quickly consumed. Generally, it has been suggested that microorganisms have little effect on the molecular weight distribution of the main polymer peak and that only abiotic factors could substantially alter this distribution^36,37^. Whereas, others have claimed a reduction in the number-average molecular weight (M_n_)^24,35^, this effect has also been observed in this study. The appearance of a tail in the lower molecular weights for the microbially treated sample resulted in the decrease of the M_n_ and suggests that some polymer chain scission takes place leading to the formation of smaller fragments. This is consistent with the increase of the intensity of the peak attributed to the vinyl functional groups observed in the FTIR spectra^38^ of the PS films exposed to the BIOG community. A decrease in the intensity of bands corresponding to double bonds was noticed in case of PS treated with INDG community, which is also suggested as indirect evidence of biodegradation^24^. Under natural conditions, the biofilm network induce changes on the polymer’s surface altering the synthesis of the functional group on the substrate. Polymer samples showed signs of bio-erosion since a rough surface with a lot of fissures was detected for the PS pieces subjected to microbial attack. Instead the weathered PS pieces have smooth surfaces without any significant cracks or holes.

In general, plastics provide a niche for marine micro- and macrofoulers, thus a diverse group of organisms rapidly colonize (within 24 h) them in a substratum as well as spatial and seasonal manner^11,27^. The acclimated indigenous and bioaugmented marine communities developed a dense biofilm already from the first month as visualized with scanning microscopy, while high adhered cell densities were recorded throughout the experimental period. Moreover, the population at the end of phase II preferred the sessile way of lifestyle (exhibiting reduced motility). Limited nutrient concentrations can favor interspecies competitive interactions developed through many different strategies^39^. Generally, highly adhesive cells have an advantage over the less adhesive cells when the substratum serves as a nutrient source^40^. The investigation of microbial succession on PS films revealed a time depended pattern, where the biofilm communities were clustered by age. Likewise, the composition of young marine bacterial communities adhered to polystyrene or granite rock is similar and differs with the old communities^41^. The type of starting inoculants could not separate the biofilm communities, indicating that the polymer may be a significant factor that shapes the plastic associated communities. Similar results were also obtained when the PE successional dynamics were assessed^42^. Moreover, biofilm microbial communities on six different coating including polystyrene shared a significant number of common OTUs but the composition depended on the type of coating^43^. Analysis of the biofilm microbiome at the end of phase I and II with high next-generation technologies revealed again a temporal separation, where the non-acclimated and acclimated assemblages were distinct.

Members of Alphaproteobacteria and Gammaproteobacteria were highly enriched in the acclimated assemblages. These groups have been also detected in “plastisphere” of plastic litter collected in the North Pacific Gyre^14^, while Alphaproteobacteria dominate the polystyrene associated microbiome in Hong Kong coastal waters^44^. Gammaproteobacteria have also been reported to be strongly associated with low-density polyethylene (LDPE)^12^ and polyethylene terephthalate (PET)^17^. Few biomarker species were detected when the non-acclimated and acclimated INDG biofilm populations were compared. It can be speculated that the increase or decrease in the abundance of some species contributed to the highest weight reduction recorded at phase II. To support, the abundance of plastic associated genera^21,45,46^ such as *Pseudomonas* and *Flavobacterium* are increased in INDG acclimated communities. Members of genera that have previously highlighted for their ability to degrade plastics^31,47,48^, have been also enriched in these communities. Interestingly, the majority of the phase II-enriched OTUs are known hydrocarbon degraders or they tend to thrive in oil polluted habitats. For example, species of the genera *Thalassospira* and *Alcanivorax* have been found able to proliferate in hydrocarbon polluted environments and bear catabolic genes responsible for hydrocarbon degradation^49–52^. In accordance, high numbers of *alkB* gene were detected within biofilm assemblages throughout phase II. Moreover, PICRUSt analysis predicted the significant increase of genes participating in xenobiotic and styrene degradation in these communities.

## Conclusions

In the present study, PS degradation by marine consortia was assessed in a holistic approach, since unrevealing the mechanisms beyond polymer degradation after their immersion in oceans could aid towards sustainable management solutions. Methods describing polymer characteristics were employed and the results suggest induced chain scission since a decline in the number-average molecular weight together with alteration in the intensity of double bonds on PS films was demonstrated. Complementary, signs of bio-erosion were revealed with scanning electron microscopy. The plastic associated microbiome developed in a time dependent pattern towards efficient microbial network able to degrade PS films under simulated natural conditions. Functional analysis predicted sessile adhered communities enriched with hydrocarbon and xenobiotic degradation genes.

## Methods

### Polystyrene collection

Polystyrene pieces, which were previously exposed to natural weathering, were collected form sandy beaches in Northern Crete; Agios Onoufrios (coordinates: 35.549128, 24.061855) and Kalathas (coordinates: 35.554538, 24.085120) in Chania, Greece. The pieces were selected based on the polymer identification symbols scheme and, thus only pieces with the number 6 enclosed by the three “chasing arrows” triangle were selected and transferred to the lab. Next, they were cleaned with water and soap and sterilized with 70% ethanol solution overnight. The PS items were dried at 50 °C for 24 h and then cut in 1 cm^2^ surface area pieces. After weighting, they were strung from a fishing line. In total 6 fishing lines were put in every beaker in accordance with the sampling months and 5 PS pieces were hanged from every line (n = 15 for each treatment). A combination of the string number with the position of a piece along the string, allowed the identification of each piece of plastic. The initial weight of PS items was approximately 45 mg.

### Biodegradation assays

A two-phase microcosm experiment was conducted in sterilized beakers with polystyrene as the sole carbon source. During the phase I, the indigenous marine community alone or bioaugmented with strains able to grow with PS as the sole carbon source were incubated with the sterile PS pieces for 6 months in enriched filtered saline water (C:N:P ratio of 100:10:1). At the end of each month, one fishing line from every replicate was permanently removed for analysis. At the end of month 6, the biofilm cells were harvested and stored in glycerol solution at −80 °C. For the phase II, the sterile PS pieces were incubated with the acclimated communities under same conditions.

### PS competent bacteria and consortia development

Competent strains able to use PS as sole carbon source and affiliated with the genera *Rhodococcus*, *Shewanella* and *Pseudomonas* were provided from Prof. Corvini’s lab (University of Applied Sciences and Arts, Switzerland). *Shewanella* sp. was isolated from water and plastic samples taken under the cages of a fish farm, *Pseudomonas* sp. was isolated from a surface water sample taken from Nordnes, Norway and *Rhodococcus* sp. was isolated from seawater samples taken at Korsfjorden/Bjørnefjorden, Norway. The strains stem from successive enrichment cultures in where polystyrene was the only available carbon source.

The strains and the acclimated biofilm communities were cultured overnight in Standard I nutrient broth (7.8 g peptone from meat, 7.8 g peptone from casein, 2.8 g yeast extract, 5.6 g NaCl and 1 g glucose per 1000 ml distilled water) at 28 °C under continuous shaking (120 rpm) and were harvested at the late log phase. They were further washed with sterilized NaCl solution (8.5 g L^−1^) and inoculated (initial concentration: 1 × 10^8^ CFU ml^−1^) to the different treatments.

When only the indigenous marine community was inoculated, the treatments were identified as “indigenous (INDG)”,while when the indigenous marine community was supplemented with the PS competent strains the treatments were identified as “bioaugmented (BIOG)”.

### Weight reduction

At the end of each month, the bacterial biofilm was removed from all the PS pieces belonged to the same fishing line and the flakes were further washed and dried at 50 °C for 3 days in order to measure the weight. The degradation was estimated by calculating the percentage of weight loss from the initial measurements in a balance with a 6-digit accuracy.

### Scanning electron microscopy (SEM)

The monitoring of the erosion on the surface and the biofilm development on the PS pieces was visualized under SEM, as previously described^42^.

### Estimation of growth of free and biofilm bacteria

Water samples and biofilm samples taken by scratching the polymer surface were serially diluted and cultured on plates with Standard I medium. They were incubated for 7 days at 20 °C and the number of colonies was enumerated. Samples were collected at the end of the phase I and every month during phase II.

### Gel permeation chromatography (GPC)

The number and weight average molecular weight (M_n_ and M_w_ respectively) of PS samples were determined by GPC (Waters). The instrument was equipped with a Waters 515 pump, two PL mixed-D and mixed-E columns and a Waters 410 refractive index detector operating at 35 °C. Calibration was based on a series of six narrow MW linear polystyrene standards with molecular weights ranging from 580 to 578,500 g mol^−1^. THF was used as the eluent at a flow rate of 1 ml min^−1^.

### FTIR

The functional groups of the polystyrene films were detected with an Attenuated total reflectance – Fourier transform infrared spectroscopy (FTIR). A Frontier FT-IR spectrometer (PerkinElmer, Waltham, Massachusetts, USA) was used and the spectra were obtained and processed using PerkinElmer’s Spectrum software. The absorbance values ranged from 4000 cm^−1^ to 650 cm^−1^ with 4 cm^−1^ scan resolution. Background scans for the reflectance of the surrounding atmosphere were performed before each sample scan and the sample’s peak heights were obtained by performing a baseline correction, subtracting the background spectrum from the sample spectrums.

### Biofilm community structure

Bacterial genomic DNA was isolated from the biofilm of at least three PS pieces belonged to the same replicate of each treatment, pooled and eluted in TE buffer. Samples were collected at the end of the first phase and at the end of every month during the second phase to monitor the development of the biofilm communities. DNA was extracted according to the CTAB protocol for the extraction of bacterial genomic DNA.

Automated rRNA intergenic spacer analysis (ARISA) was performed using the primers ITSF (5′-GTCGTAACAAGGTAGCCGTA-3′) and ITSReub (5′-GCCAAGGCATCCACC-3′)^53^ for the amplification of the ITS1 region in the rRNA operon plus ca. 282 bases of the 16S and 23S rRNA, as previously described^42^.

In order to perform the metagenomic analysis the DNA concentration was determined using the Quantifluor dsDNA assay (Promega Corporation, USA). The concentration of the amplicons were measured and adjust to an equimolar amount of 4 nM before sequencing. Next generation sequencing of 16S rDNA genes amplified from DNA extractions were performed according to Illumina’s application note (part #15044223, Illumina, San Diego, USA). Primers for sequencing were 515 F (5′-GTG CCA GCM GCC GCG GTA A-3′) and 806 R (5′-GGA CTA CHV GGG TWT CTA AT-3′). PCR steps were performed using the KAPA HiFi HotStart kit (Kapa Biosystems, Wilmington, USA). The thermocycler program was the following: 95 °C for 3 minutes, followed by 25 cycles of 95 °C for 30 seconds, 55 °C for 30 seconds and 72 °C for 30 seconds, respectively, with a final elongation step at 72 °C for 5 minutes. The completed DNA libraries were run on the MiSeq Illumina, using a MiSeq Reagent Kit v3 (600-cycle). The sequences were deposited in BioProject (PRJNA378706), the Submission ID is SUB2440072.

The abundance of *alkB* gene in the adhered assemblages was monitored during phase II using a StepOne Plus System (Applied Biosystems Inc., Foster City, CA, USA). The primer pair alkB-f (5′-AAYACIGCICAYGARCTIGGICAYAA-3′) and alkB-r (5′-GCRTGRTGRTCIGARTGICGYTG-3′)^54^ was used while qPCR master mix and conditions are performed as previously described^55^. The amplification efficiency was 105%, the amplification coefficient (R^2^) was 0.98, while melting-curve and 1.5% agarose gel were used for checking the specificity of the products.

Functional predictions were performed using a database of phylogenetically referenced genomes (PICRUSt, Phylogenetic Investigation of Communities by Reconstruction of Unobserved States^56^). This bioinformatics tool connects the taxonomic classification (exploiting the 16 S rRNA gene) with metabolic capabilities. Function predictions were categorized on the Kyoto Encyclopedia of Genes and Genomes (KEGG) classification at level 3.

### Statistical analysis

Statistical analysis was carried out with the automatic R^57^. Two way ANOVA was applied to the data in order to evaluate the effect of month or treatment to the different studied variables.

The analysis of ARISA fragments was performed with the Bioanalyzer software, fragments with size less or equal to 2 bp were considered identical and a minimum peak height of 150 fluorescence units was considered for further analysis^58^. Fragments less than 150 bp were removed. The analysis of the OTU table was performed by Primer6 software. A resemblance matrix with the normalized (presence/absence) values was analysed using the Bray-Curtis similarity method and clustered in the complete linkage mode to generate a dendrogram based on per cent similarity while the degree of similarity was explored with the permutation-based hypothesis statistical test ANOSIM.

The paired-end reads were assembled with PANDAseq version 2.8^59^ and QIIME package, version 1.9.1^60^ was used for downstream analysis. Briefly, sequences were picked *de novo*, using the Greengenes database updated in May, 2013 (http://greengenes.lbl.gov) with a 97% identity threshold. Rarefied OTU tables were generated and all samples were subsampled to 1629 sequences per sample Next, a PCoA plot was performed on the unweighted UniFrac distance matrices, using R^57^ (package “phyloseq.^61^”); the ANOSIM statistical test was calculated in QIIME.

The Linear Discriminant Analysis Effect Size (LEfSe)^62^ was also performed in QIIME in order to elucidate OTUs with different abundances between the acclimated and non-acclimated biofilm communities. The p-value was set at 0.05 and the LDA log score threshold at 4.

### Data Availability

All data generated or analyzed during this study are included in this article (and its Supplementary Information files). The datasets generated during the current study are also available from the corresponding author upon reasonable request.

## Electronic supplementary material

Supplemental information
