# Supplementary material for: Biodegradation of weathered polystyrene films in seawater microcosms
Source: Sci Rep. 2017 Dec 21;7:17991. doi: 10.1038/s41598-017-18366-y (PMC5740177; doi:10.1038/s41598-017-18366-y)
Supplement: Supplementary file 1 — Supplemental information [file 41598_2017_18366_MOESM1_ESM.pdf]

Biodegradation of weathered polystyrene films in seawater microcosms

Evdokia Syranidou[1], Katerina Karkanorachaki[1], Filippo Amorotti[1,5], Martina Franchini[1], Eftychia Repouskou[2], Maria Kaliva[3], Maria Vamvakaki[3], Boris Kolvenbach[4], Fabio Fava[5], Philippe F-X Corvini[4] and Nicolas Kalogerakis[1*]

[1] School of Environmental Engineering, Technical University of Crete, Chania, Greece
[2] School of Mineral Resources Engineering, Technical University of Crete, Chania, Greece.
[3] Department of Materials Science & Technology, University of Crete, Heraklion, Greece
[4] Institute for Ecopreneurship, School of Life Sciences, FHNW, Switzerland
[5] Department of Civil, Chemical, Environmental and Materials Engineering (DICAM), University of Bologna, Bologna, Italy

SUPPLEMENTARY INFORMATION

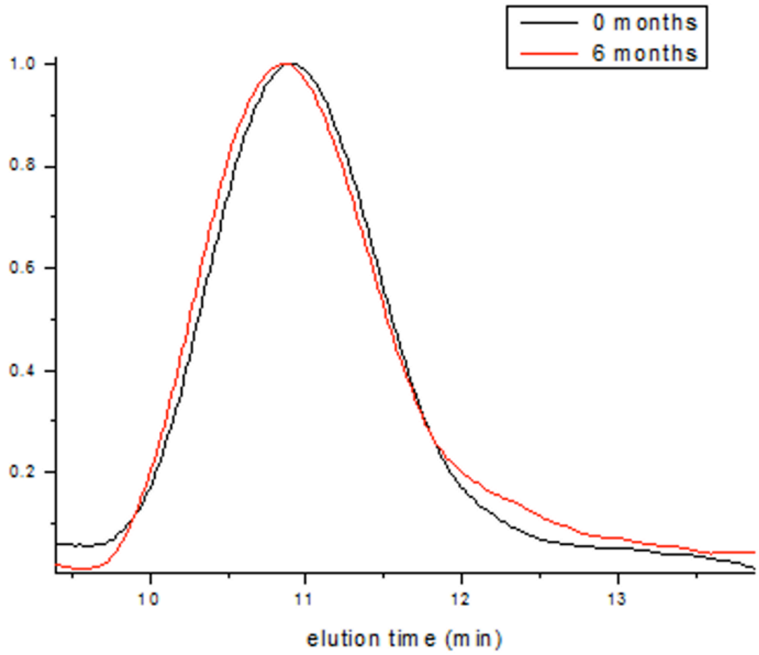

Figure S1. Gel permeation chromatography (GPC) profile of microbially treated PS films initially and at time 6 months.
